# Small is big: growing impact of small molecule mass spectrometry in infectious disease drug development

**DOI:** 10.1128/msphere.00860-25

**Published:** 2026-04-29

**Authors:** Laura-Isobel McCall

**Affiliations:** 1 Department of Chemistry and Biochemistry, San Diego State University 7117, San Diego, California, USA; Virginia-Maryland College of Veterinary Medicine, Blacksburg, Virginia, USA

**Keywords:** drug development, mass spectrometry, infectious disease

## Abstract

Small molecule analysis by mass spectrometry (MS) in drug discovery and development has historically focused on confirmation of synthesis products and on drug quantification in pharmacokinetic and pharmacodynamic studies. However, advances in sensitivity, throughput, and cost of mass spectrometers, alongside improvements in data analysis pipelines, have led to increasing contributions of small molecule MS across drug discovery and development stages. In this minireview, I will discuss these recent technical advances in the context of infectious disease drug discovery and development, highlighting applications to high throughput screening and hit identification, the discovery of novel mechanisms of action, personalized treatment and diagnostics, and fighting treatment failure.

## INTRODUCTION

In my 2021 mSphere of Influence commentary (1), I discussed three very different manuscripts: a study on Chagas disease patient perspectives, reflecting critical gaps in care (2), a new 3D metabolomics approach to understand *in situ* metabolic effects of infection (3), and the potential of metabolic interventions to induce disease tolerance in a context-specific manner (4). A common thread across these papers has been inspiring me to use new mass spectrometry (MS) and metabolism-centric approaches to rethink drug discovery and development, particularly in the context of infectious diseases.

Standard infectious disease drug discovery is divided between targeted approaches, where the target of interest is identified at the start of the workflow, and phenotypic drug discovery, where the specific target is not pre-determined and instead readouts focus on aspects such as pathogen killing. Usually, thousands of molecules are screened for the desired activity in a high throughput fashion, with cross-screening or filtering based on safety metrics (5). Current artificial intelligence (AI)-powered virtual screens use a similar workflow, but with these initial screens taking place *in silico* (6, 7). Further development refines initial hit structures with regards to activity, safety, and pharmacokinetics/pharmacodynamics (PK/PD) until compounds can be tested in humans (8). Clinical testing follows a standard workflow of phase 1–3 testing with a priority on safety and appropriate dosing, followed by efficacy readouts. This structure has been in place for more than 60 years (9, 10). The often-cited cost for drug discovery and development exceeds $1 billion when failures are included, although this is highly variable depending on the indication and may be lower for infectious disease therapeutics (11). Clinical development alone takes a median of more than 8 years, with longer timelines for antibacterials than antivirals (12).

**Fig 1:**
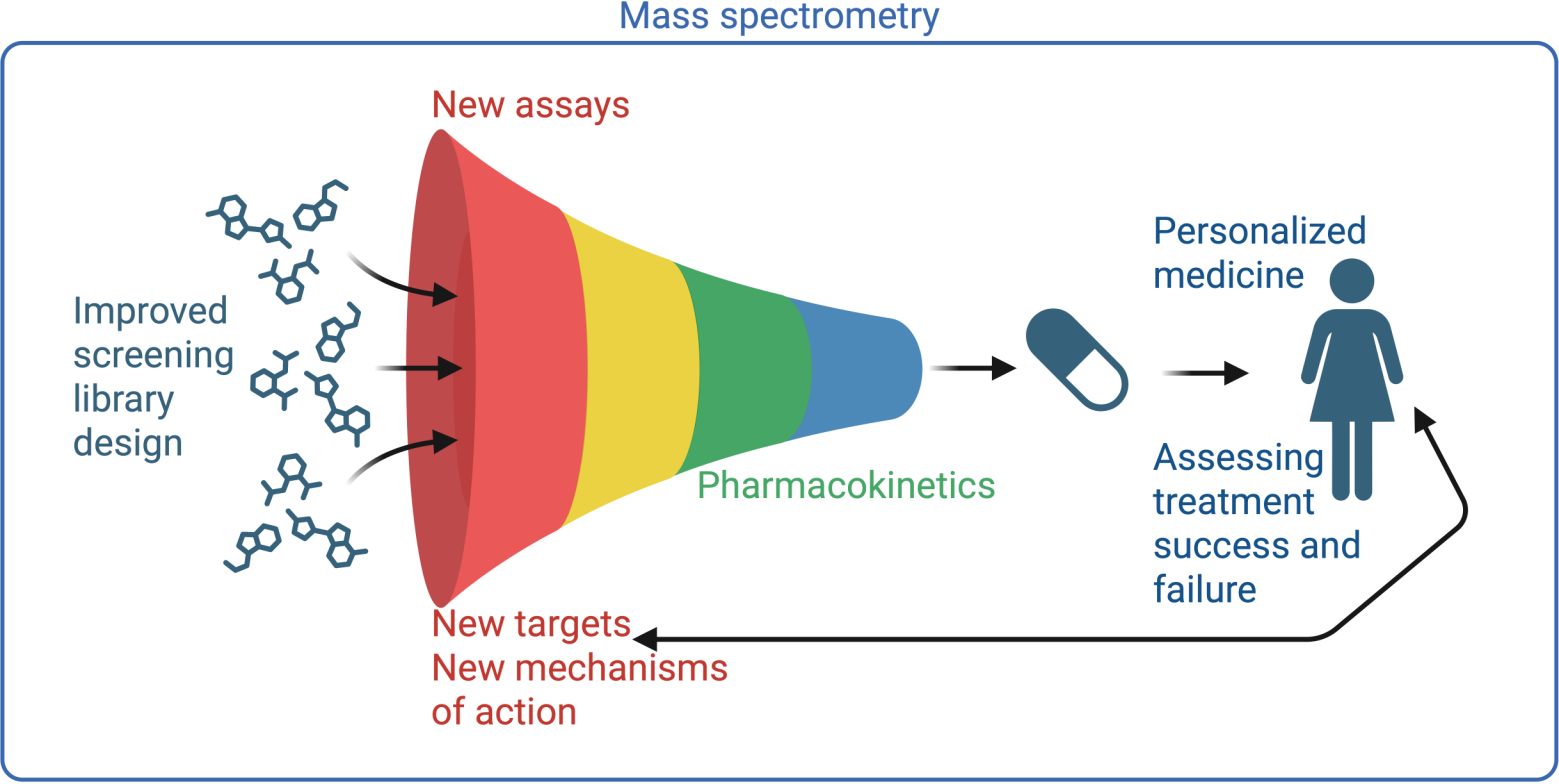
Applications of small molecule MS throughout the drug discovery and development pipeline. Created in BioRender.

The field of drug discovery and development is ripe for innovation. Indeed, the last several years have provided major advances, most recently the great potential of AI. Nevertheless, the quality of the output from AI is highly dependent on the availability of quality input and still currently necessitates expert verification (7). Thus, experimental science remains a key component of drug discovery. MS provides quantitative and qualitative data on molecule size, molecular formula, molecule structure, and molecule abundance. It can be applied to a considerable range of molecules, which are broadly divided in drug discovery and development between large molecules (particularly proteins; biologics and biosimilars) vs small molecules less than ~1,500 Da in size. Small molecule MS plays critical roles in initial compound screens, in pharmacokinetics/pharmacodynamics (PK/PD) considerations, and with emerging roles in precision medicine (Fig. 1). In this Full Circle minireview, I discuss the role of new technical and conceptual advances in this field to expedite infectious disease drug discovery and development.

## STARTING AT THE BEGINNING: SMALL MOLECULE-BASED APPROACHES TO EXPEDITE HIGH THROUGHPUT SCREENING AND HIT IDENTIFICATION

Almost all modern small molecule drug discovery begins by screening small molecule collections for interactions with targets of interest, or for a desired phenotypic effect. This process has been revolutionized by new artificial intelligence (AI) developments, initially through AlphaFold 2’s ability to predict protein structures (13), augmented in AlphaFold 3 with the ability to predict small molecule ligand docking (14). This makes virtual screens possible, even potentially for microbial target proteins for which structures have yet to be experimentally determined, with the caveat that output quality will depend on the confidence of the structural prediction and that binding does not necessarily equate inhibition.

However, these AI-powered screens are usually implemented on known small molecule structures. Virtual libraries may not be enriched in the complex stereochemistry that may be a better fit for 3D pockets on targets (15). Furthermore, very large virtual screens lead to a high absolute number of artefactual hits, which is problematic given that budgets for synthesis and hit-to-lead are usually fixed (16). Natural product-based drug discovery still plays a key role in the identification of novel bioactive chemotypes (17). MS has enabled major advances in natural product library design in the last five years, by prioritizing extract selection (18–22) and by enabling “dereplication”: the discrimination between previously discovered and novel chemotypes (23). Many natural product screening libraries are built from extracts that come from related biological sources (e.g., a library of plant extracts). Because of these related biological origins, they will share common molecules so that multiple library wells will have overlapping chemical composition within a library or with previously tested libraries. Spectral similarity can be leveraged to prioritize extracts with the least chemical overlap even if the structure of the molecules present in the library is not yet known (18). Jointly, these advances will enable smaller-sized screening campaigns, shrinking from the prior hundreds of thousands or millions of tested extracts (24) to a few thousand or less (18). The resulting cost reduction can be leveraged to implement more costly but more disease-relevant screening systems, moving beyond biochemical assays on purified proteins to complex activity assays and complex systems such as organs-on-a-chip (25). Examples of this latter approach are optimized primary human hepatocyte or induced pluripotent stem cell (iPSC)-derived liver organoid systems to study *Plasmodium vivax* liver stages and assess anti-hypnozoite activity (26, 27). More relevant models are critical since up to half of infectious disease drug candidates fail to advance from phase II to phase III or from phase III to approval (28, 29).

In the case of target-based drug discovery, selection of the protein to target is another critical initiating step in drug discovery campaigns. This is usually a multifactorial decision process, in which small molecule MS can play a role. In theory, the identification of a unique metabolite produced by a microorganism and its associated metabolic pathway should represent a good target for drug discovery. However, due, in part, to the long drug development timeline and the later emergence of modern metabolomic techniques compared to the “Golden Age of Antibiotics,” most metabolism-targeting therapeutics were discovered serendipitously rather than intentionally (e.g., isoniazid and *Mycobacterium tuberculosis* mycolic acid synthesis [30]). Other obstacles include differences in pathogen metabolism between cultures and *in vivo* (e.g., [31]), worsened by inconsistent media composition between batches (32), and the focus of many metabolomic studies on easy-to-annotate metabolites, which effectively leads to the identification of changes in metabolic pathways well characterized in humans rather than novel microbial metabolites. The issue of metabolic inconsistency between *in vitro* and *in vivo* conditions is being addressed by newer sensitive low-input and spatial metabolomics techniques that will enable the discovery of metabolic pathways operating in true *in vivo* conditions (33–35), and the increasing development of culture conditions that better replicate the *in vivo* nutritional environment (36–38). Novel microbial metabolite discovery is facilitated by collaborations with natural product researchers who are used to structure elucidation from microbial sources (39). The discovery of structural novelty will also be accelerated by the development of new metabolite annotation and structure elucidation tools that go beyond matching to existing metabolite libraries. New tools developed in this context include MSNovelist (40), DiffMS (41), a new version of ICEBERG (42), and OMG (43). Novel pathway elucidation tools such as MEANtools will also be helpful (44). Overall, a key barrier for implementation in drug discovery is currently the accuracy of the top prediction(s) by these tools, but continued advances in artificial intelligence, growing public data sets, and the MassSpecGym benchmark (45) will help address this challenge.

Once a target has been selected, small molecule MS also plays a key role in MS-based enzyme assay readouts, quantifying enzymatic reaction products in high throughput and with the potential to enable complex multifactorial readouts, for example, of metabolism renormalization in the context of disease tolerance (46, 47). Compared to bioluminescence or fluorescence readouts, MS-based assays directly detect enzymatic activity, with less risk of confounders such as fluorescence interference, and enable expansion beyond existing reporter systems (48). Recent advances include direct coupling of acoustic ejection of samples (e.g., from an enzymatic activity assay) with electrospray ionization (ESI)-MS to measure reaction products and thus the inhibition of this reaction to identify hits. Throughput of these methods exceeds 80,000 activity assays per day (49, 50). High throughput of over 200,000 wells per day can be achieved with new desorption electrospray ionization (DESI)-MS methods (51). While Matrix-Assisted Laser Desorption/Ionization (MALDI)-MS usually requires additional steps for matrix addition and may suffer from matrix interference with smaller enzymatic reactants or products, it too can be suitable for high throughput screens (52). In cases where assay buffers or other assay conditions may interfere with MS, RapidFire solid phase extraction systems coupled to MS are a powerful system. RapidFire–MS high throughput screening has been recently used to identify inhibitors of SARS-CoV-2 nonstructural protein 14 and *Trypanosoma cruzi* M17 leucyl-aminopeptidase, for example (53, 54).

Phenotypic assays using MS readouts are more rare, but a notable example includes identification of compounds that reverted the lipid and metabolite profile indicative of a macrophage pro-inflammatory phenotype (55). These profiles can also be used to identify candidate combination regimens by integration with metabolomic analyses of gene knockouts, as performed in *Escherichia coli* (56). Conversely, identifying targets of hits from phenotypic screens can be challenging. Recent work has compared the metabolic impact of these hits to the profile caused by inhibitors of known pathways, to enable target identification, for example, in *Plasmodium falciparum* (57) or in *Mycobacterium tuberculosis* (58). This is also important to rule out inhibitors of known or promiscuous targets, for example, cytochrome *b* in *Trypanosoma cruzi* (59). More traditional approaches perform metabolomic analysis and identify specific individual or pathway-level metabolic changes, which, in combination with other techniques such as selecting for resistant mutants, can identify the molecular target (60).

## BEYOND ANTI-MICROBIALS: METABOLISM-TARGETING THERAPEUTICS TO ADDRESS DISEASE SYMPTOMS

Such novel metabolism-centric and MS-driven assays will also be key to enable screening assay readouts that are not merely based on pathogen numbers but instead use functional readouts. As demonstrated by the plethora of post-infectious conditions (61, 62), many infectious diseases are associated with clinical symptoms that persist or worsen after the pathogen is cleared by the immune system or treatment. These conditions are, therefore, not treatable by conventional antimicrobial therapeutics. In Chagas disease, antiparasitic treatment does not prevent mortality if administered in advanced symptomatic disease (63). We demonstrated that manipulating metabolism is sufficient to prevent acute-stage mortality in mouse models of Chagas disease: treatment with carnitine improved cardiac function and re-normalized cardiac metabolism in the absence of anti-parasitic or immunomodulatory effects (47). Conversely, improved tissue or biofluid metabolic renormalization, as measured by small molecule MS, can be used as a criteria to evaluate therapeutic approaches for their ability to outperform the current standard-of-care (64, 65). For example, metformin is being evaluated to treat long COVID, and in combination with anti-mycobacterials, to treat tuberculosis. These treatments were designed based on metformin’s effects on mitochondrial complex I and glucose levels and its anti-mycobacterial effects (66–70). Metabolomic analyses in this context could serve as an objective measure of the efficacy of metformin treatment, by determining whether it re-normalizes infection-associated metabolic dysregulation. Importantly, these new treatment approaches are targeting host pathways and, thus, most likely do not select for pathogen resistance. Given the overlap in metabolic impact between multiple infectious diseases (71–73), this may represent an opportunity to develop multifunctional treatments, reducing drug development costs. While metabolic plasticity, metabolic redundancy, off-target, and toxic effects on other organs or cell types will naturally be a concern, the progress on metabolic modulators in cancer support feasibility (74, 75).

## PRIORITIZING CARE: ADVANCES IN PERSONALIZED MEDICINE AND PATIENT IDENTIFICATION IN INFECTIOUS DISEASES

Advances in infectious disease diagnostics are helping to quickly differentiate between bacterial and viral causes, identify the specific responsible agents, and determine their susceptibility to existing drugs to prioritize treatment. Major MS-based advances in that context include Matrix-assisted laser desorption/ionization (MALDI)-based techniques to identify bacteria in clinical samples, even without culture (76, 77), and a metabolic preference assay using liquid chromatography-MS that can differentiate between the most common causative bacterial agents for bloodstream infection and determine their antibiotic susceptibility profile in less than 20 h (78). Likewise, small molecule analysis can rapidly determine whether urinary tract infection symptoms are actually caused by a bacterial infection, thus guiding subsequent treatment decisions (79). A similar approach can differentiate between self-limiting dengue fever and more severe dengue hemorrhagic fever (DHF) or the potentially fatal dengue shock syndrome (DSS) (80) and between post-treatment Lyme disease symptoms/syndrome and patients lacking this condition (81). Small molecule biomarkers can also predict which people infected with *Mycobacterium tuberculosis* will advance to severe symptomatic disease and which will remain asymptomatic (82, 83). Small molecule MS has also been used to elucidate mechanisms of disease exacerbation in cystic fibrosis from sputum samples (84). These approaches can help prioritize treatment delivery or facilitate treatment compliance in high-risk populations. For example, identification of people at risk to progress to active tuberculosis among household contacts of tuberculosis cases could prioritize them for isoniazid prophylaxis (82, 83, 85, 86). A similar approach could be useful for any infectious condition where current treatment adverse effects are high and rates of progression to severe disease are moderate. Coupled with the long treatment regimens, a biomarker that could predict treatment response at the time of treatment onset would be highly effective.

## UNDERSTANDING TREATMENT FAILURE AND CREATING NEW DEFINITIONS OF TREATMENT SUCCESS

Conversely, small molecule analysis can be used to understand why a treatment regimen was unsuccessful. Treatment failure can be divided into two mechanisms: failure to clear a pathogen, and failure to improve symptoms (87). Small molecule MS analyses have been a mainstay technique for PK/PD studies in the drug discovery pipeline. Historically, PK studies have been performed in healthy uninfected animals due to logistical ease. In the extreme case of tubercular granulomas, this is problematic since drug penetration into the granuloma differs from drug distribution in healthy tissue (88). In cutaneous leishmaniasis, miltefosine presents with an opposite pattern, accumulating in the granuloma but found at lower levels in surrounding fibrotic tissue, where parasites persist (89). However, even in cases where structural rearrangements are more minor, different drug distribution may be observed based on infection status and tissue damage. For example, our work revealed differential drug accumulation between uninfected mice and mice infected with *Trypanosoma cruzi*. Cardiac tissue ketamine and ketamine metabolite levels were correlated to the degree of inflammation and cardiac fibrosis (90). Likewise, *S. aureus* infection changed PK parameters for tedizolid and linezolid (91).

Plasma exposure and free plasma drug levels are usual PK readouts (92) but are only an indirect and sometimes inaccurate surrogate for drug accumulation in the right target organs, target cells, and target organelles. Technical developments in single-cell MS and fine spatial resolution MS imaging now enable subcellular analyses of drug levels. Single-cell analyses have been used in the context of cancer therapy to quantify drug and drug metabolite levels in single cells, a method which by extension will be applicable to infectious diseases to quantify drug levels inside individual infected cells (93, 94). With progress in phagosomal, single-organelle, and single-vesicle MS techniques (35, 95), the achievable scale is now approaching what is needed to quantify drug levels in individual eukaryotic protistan pathogens. MS imaging and other spatial approaches can reveal poor drug penetration at specific tissue sites, for example, into tuberculosis granulomas (88, 96) or at the base of the lung (3). Longer-term monitoring can also reveal the kinetics of drug accumulation and clearance; a drug that accumulates or clears slowly may lead to selection of drug-resistant mutants, depending on whether the dose is insufficient to kill every local microorganism, the pathogen is capable of dormancy or latency, and resistance is pre-existing or emerges during the course of treatment (97, 98).

Small molecule MS is also useful to understand mechanisms of post-infectious conditions and failure of existing treatments to improve disease symptoms. Lack of metabolic re-normalization post-treatment can serve as an indicator of treatment failure and an opportunity to identify new targets for intervention (64, 65). In turn, metabolic renormalization can serve as a surrogate endpoint to evaluate treatments that seek to promote disease tolerance, for example, by restoring normal metabolism (47) or promoting normal organ function (99). Such approaches are showing potential to treat infections but necessitate alternative mechanisms to monitor treatment success since assessment of their efficacy cannot rely on pathogen load measurements. Small molecule predictors of disease symptom progression may be useful in this context.

## CONCLUSION

Drug development failure rates remain unacceptably high, driving up drug development costs and timelines (92). Small molecule MS has the potential to expedite drug discovery and development while reducing costs. MS-based methods that enable the discovery of new disease-relevant targets and facilitate pre-clinical testing under conditions that are better replicates of *in vivo* disease pathogenesis, coupled with better ways to stratify and prioritize patients for treatment, will reduce failures from lack of efficacy. Better assessment of drug distribution under infection-relevant conditions will improve both efficacy and safety. Lowering failure rates and shortening timelines to the clinic will lead to reduced drug development costs, which can then be used to reduce the financial burden of treatment on patients. In parallel, these may help improve incentives for drug development in the context of diseases of poverty, where the need is great but revenue potential is low. Overall, patient needs for better treatments, better diagnoses, and better access have been motivating fundamental research to expedite drug discovery and development in a way that is responsive to the needs of the infectious disease clinical and patient community.
